# Ultrahigh resolution MS^1^/MS^2^-based reconstruction of metabolic networks in mammalian cells reveals changes for selenite and arsenite action

**DOI:** 10.1016/j.jbc.2022.102586

**Published:** 2022-10-09

**Authors:** Teresa W.-M. Fan, Qiushi Sun, Richard M. Higashi

**Affiliations:** 1 Center for Environmental and Systems Biochemistry (CESB), University of Kentucky, Lexington, Kentucky, USA; 2 Department of Toxicology and Cancer Biology, University of Kentucky, Lexington, Kentucky, USA; 3 Markey Cancer Center, University of Kentucky, Lexington, Kentucky, USA

**Keywords:** selenite, arsenite, [^13^C_6_]-glucose, [^13^C_5_,^15^N_2_]-glutamine, positional isotopologues, stable isotope resolved metabolomics, metabolic pathway reconstruction, BAsT, arsenite-transformed BEAS-2B cells, F6P, fructose-6-phosphate, GOT, glutamic-oxaloacetic transaminase, HBP, hexosamine biosynthesis pathway, IC-UHR-MS^1^/DI-MS^2^, ion chromatography-ultrahigh resolution-MS^1^/data independent-MS^2^, ME, malic enzyme, MS, mass spectrometry, PC, pyruvate carboxylase, PDH, pyruvate dehydrogenase, PPP, pentose phosphate pathway, R5P, ribose-5-phosphate, Ru5P, ribulose-5-phosphate, S7P, sedoheptulose-7-phosphate, SIRM, stable isotope–resolved metabolomics, TALDO, transaldolase, TKT, transketolase

## Abstract

Metabolic networks are complex, intersecting, and composed of numerous enzyme-catalyzed biochemical reactions that transfer various molecular moieties among metabolites. Thus, robust reconstruction of metabolic networks requires metabolite moieties to be tracked, which cannot be readily achieved with mass spectrometry (MS) alone. We previously developed an Ion Chromatography-ultrahigh resolution-MS^1^/data independent-MS^2^ method to track the simultaneous incorporation of the heavy isotopes ^13^C and ^15^N into the moieties of purine/pyrimidine nucleotides in mammalian cells. Ultrahigh resolution-MS^1^ resolves and counts multiple tracer atoms in intact metabolites, while data independent-tandem MS (MS^2^) determines isotopic enrichment in their moieties without concern for the numerous mass isotopologue source ions to be fragmented. Together, they enabled rigorous MS-based reconstruction of metabolic networks at specific enzyme levels. We have expanded this approach to trace the labeled atom fate of [^13^C_6_]-glucose in 3D A549 spheroids in response to the anticancer agent selenite and that of [^13^C_5_,^15^N_2_]-glutamine in 2D BEAS-2B cells in response to arsenite transformation. We deduced altered activities of specific enzymes in the Krebs cycle, pentose phosphate pathway, gluconeogenesis, and UDP-GlcNAc synthesis pathways elicited by the stressors. These metabolic details help elucidate the resistance mechanism of 3D *versus* 2D A549 cultures to selenite and metabolic reprogramming that can mediate the transformation of BEAS-2B cells by arsenite.

Metabolomics has been instrumental in accelerating the elucidation of metabolic reprogramming induced by disease states or drug treatment (1, 2, 3, 4) and the discovery of metabolism-based biomarkers (5, 6, 7). As metabolite levels are governed by many factors including rates of synthesis and degradation, multiple input and output pathways, and exchange across compartments (8), it has been challenging to reconstruct metabolic networks based on total metabolite profiles alone. Metabolic transformations through the network require numerous enzyme-catalyzed reactions that transfer the structural moiety among metabolites. Thus, the ability to track metabolite moiety will greatly reduce the ambiguities in metabolic network analysis. Stable isotope–resolved metabolomics (SIRM) fulfills this requirement by systematically tracking the transformations of individual tracer atoms from precursors to products using a combination of MS^1^ and NMR methods, which provides respectively the number and position of the tracer atoms in given metabolites. This approach has been successfully applied to determine altered metabolic activities by disease development and other perturbations in 2D/3D cell cultures (9, 10, 11, 12, 13, 14, 15), human tissues *ex vivo* (2, 16, 17), patient-derived xenograft mice *in vivo* (18, 19), and even human subjects *in vivo* (2, 20).

However, compared with mass spectrometry (MS), the moderate sensitivity of NMR limits the overall metabolite coverage. This limitation prompted us to develop an Ion Chromatography-Ultrahigh Resolution-MS^1^/data independent-MS^2^ (IC-UHR-MS^1^/DI-MS^2^) method to enable determination of tracer atom position(s) in metabolite moiety by MS with higher resolution and sensitivity than NMR. This in turn allows robust reconstruction of metabolic network responses to stressors at specific enzyme levels (21). The UHR-MS^1^ step is capable of resolving the neutron mass difference among different tracer atoms (*e.g.*, Δmass = 0.006995 amu between ^13^C and ^15^N) (10, 22). This capability enables multiplexing of biologically compatible tracer atoms such as ^13^C, ^15^N, and ^2^H in the same (*e.g.*, [^13^C_5_,^15^N_2_]-Gln) or different substrates (*e.g.*, [^13^C_6_]-glucose + [^15^N_2_]-Gln) to expand the metabolic pathway coverage while circumventing sample batch effects in multiplex SIRM studies (10, 23).

We have expanded the pathway reconstruction of purine/pyrimidine nucleotide synthesis to the reconstruction of metabolic networks consisting of the Krebs cycle, pentose phosphate pathway (PPP), gluconeogenesis, and UDP-GlcNAc synthesis pathways in 3D A549 spheroids and arsenite-transformed BEAS-2B cells. By tracing [^13^C_6_]-glucose or [^13^C_5_,^15^N_2_]-Gln transformations into the moiety of these pathway metabolites, we were able to deduce changes in specific enzyme activities induced by selenite in A549 spheroids or by arsenite in BEAS-2B cells. This information enabled us to surmise the resistance mechanism of 3D *versus* 2D A549 cultures to selenite and metabolic reprogramming that presumably mediates the transformation of BEAS-2B cells by arsenite.

## Results

Isotope enrichment distributions of major metabolites from glycolysis, the Krebs cycle, PPP, gluconeogenesis, and UDP-GlcNAc metabolism were obtained from the UHR-MS^1^ and MS^2^ spectra in both [^13^C_6_]-glucose–traced A549 spheroids ± anticancer selenite treatment and [^13^C_5_,^15^N_2_]-Gln–traced BEAS-2B cells compared with arsenite transformated BEAS-2B cells. Example MS^1^ (A) and MS^2^ (B) spectra are shown for citrate in Fig. S1. Isotopologue concentrations were calculated from the peak area ratio of samples to calibration standard mixtures after natural abundance correction, followed by normalization to the sample protein concentration.

### The Krebs cycle

The glycolytic product of [^13^C_6_]-Glc (^13^C_3_-pyruvate) enters the Krebs cycle either *via* ^13^C_2_-acetyl CoA produced from the pyruvate dehydrogenase (PDH) reaction or directly into ^13^C_3_-oxaloacetate *via* pyruvate carboxylase (PC) activity. After the first turn, the PDH-initiated Krebs cycle produces ^13^C_2_-isotopologues () of various intermediates, whereas PCB-initiated Krebs cycle generates ^13^C_3_-isotopologues () of citrate, *cis*-aconitate, malate, fumarate, and aspartate (2), and the malic enzyme (ME) reaction scrambles ^13^C in pyruvate leading to the synthesis of ^13^C_1_-metabolites () (Figs. S2*A* and 1*A*). It should be noted that this pathway scheme takes into account unlabeled carbon (●) that can come from preexisting pools of free metabolites as well as their precursors such as glycogen, proteins, and lipids.

**Figure 1:**
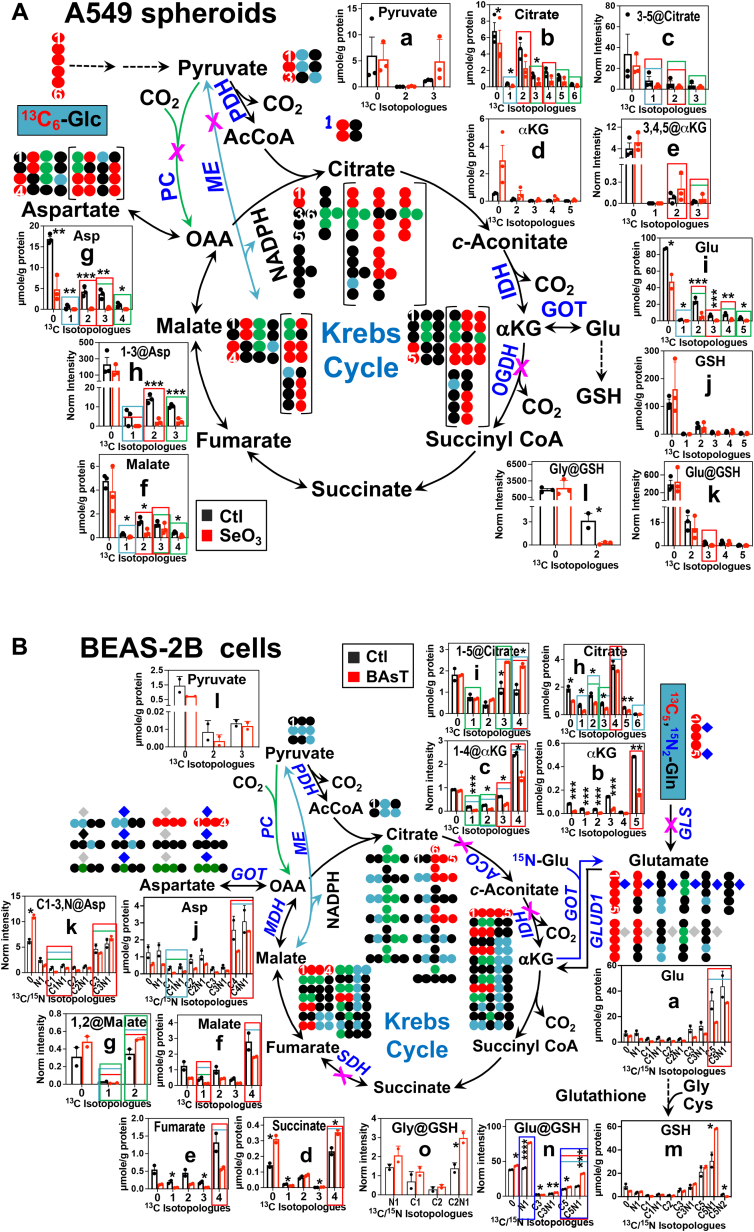
^**13**^**C and**^**15**^**N isotopologue analysis of IC-UHR-MS**^**1**^**and MS**^**2**^**data shows blocked Krebs cycle by selenite in A549 spheroids and by arsenite transformation in BEAS-2B cells.***A*, A549 spheroids. *B*, BEAS-2B cells. Polar extracts were analyzed by IC-UHR-MS^1^ and DI-MS^2^. ^13^C and ^15^N atoms were traced from [^13^C_6_]-Glc (*A*) or [^13^C_5_,^15^N_2_]-Gln (*B*) into the Krebs cycle metabolites after first and second turns (enclosed in *brackets*). Due to space limitation, not all possible labeled products are shown. ●: ^12^C; : ^14^N; : ^15^N; , , : ^13^C from the first turn of the PDH, PC, and ME-mediated Krebs cycle reactions, respectively. The X-axis refers to the number of ^13^C and/or ^15^N atoms in each isotopologue. The Y-axis represents μmole or ion intensity normalized to amounts of total protein. Data shown are mean ± SEM (n = 3) for A549 spheroids and mean ± SEM (n = 2) for BEAS-2B cells. The boxes are color-coded to denote the contribution of the GLS (*red*)/GOT (*blue*) in (*B*), PDH (*red*) in (*A*), and PC- (*green*) and ME-mediated (*light blue*) Krebs cycle reactions in (*A*) and (*B*) to given isotopologues of metabolites. αKG, α-ketoglutarate; AcCoA, acetyl CoA; ACO, aconitase; GOT, glutamate-oxaloacetate transaminase; GLS, glutaminase; GLUD1, glutamate dehydrogenase; GSH, glutathione; IDH, isocitrate dehydrogenase; ME, malic enzyme; OAA, oxaloacetate; OGDH, α-ketoglutarate dehydrogenase; PC, pyruvate carboxylase; PDH, pyruvate dehydrogenase. ∗*p* < 0.05; ∗∗*p* < 0.01; ∗∗∗*p* < 0.005; ∗∗∗∗*p* < 0.001.

In the [^13^C_6_]-Glc–traced A549 spheroids, we saw the occurrence of ^13^C_2_- (2, red box) and ^13^C_3_-citrate (3, green box), which are the respective products of PDH-initiated (canonical) and anaplerotic PC-initiated Krebs cycle (Figs. 1*A*-b and S1*A*). The presence of the ^13^C_3_-3,4,5-citrate species (3) in the MS^2^ data also points to PC activity (Figs. 1*A*-c and S1*B*). It is evident from the UHR-MS^1^ data that ^13^C_2_-citrate accumulated more than ^13^C_3_-citrate, indicating a higher activity of PDH-initiated than anaplerotic PC-initiated Krebs cycle. However, ^13^C_2_-malate (f) and -Asp (g) were comparable in levels to the ^13^C_3_-counterparts (Fig. 1*A*). This discrepancy can be accounted for by the contribution of a second turn canonical Krebs cycle activity to the ^13^C_3_ pools, which is consistent with the synthesis of ^13^C_4_-citrate (b), a specific product of the second turn. Although low in levels, ^13^C_1_-citrate and -Glu (i) were present, suggesting contribution from the ME reaction. Selenite induced the depletion of all ^13^C_2_-, ^13^C_3_-, and ^13^C_1_-isotopologues of the Krebs cycle intermediates in A549 spheroids, except for αKG (d), which showed enhanced buildup. These data are consistent respectively with inhibition of PDH, PC, and ME-mediated Krebs cycle activity, particularly at the α-ketoglutarate dehydrogenase (OGDH) step by selenite leading to the accumulation of all ^13^C-isotopologues of αKG. The ^13^C-labeling patterns of the MS^2^ fragments verified the selenite effect on PDH (2 or ^13^C_2_-1,2-Asp, h; 3 or ^13^C_3_-Glu-GSH, k) and PC (3 or ^13^C_3_-1,2,3-Asp, h) activity (Fig. 1*A*) while revealing inhibition of GSH synthesis by blocking the PDH-initiated Krebs cycle activity and Ser→Gly synthesis pathways (*cf.*, Fig. S3). The latter is evidenced by the depletion of ^13^C_3_-Glu (k) and ^13^C_2_-Gly (l) moiety of GSH. This information could not be ascertained based on the MS^1^ data of GSH (j) alone (Fig. 1*A*).

In [^13^C_5_,^15^N_2_]-Gln–traced BEAS-2B cells, the labeled Gln enters the Krebs cycle by first conversion to ^13^C_5_, ^15^N_1_-Glu (a) *via* glutaminase-catalyzed glutaminolysis and then to ^13^C_5_-αKG (b) *via* glutamic-oxaloacetic transaminase (GOT)-catalyzed transamination and/or glutamate dehydrogenase 1–catalyzed oxidative deamination. ^13^C_5_-αKG is further transformed to ^13^C_4_-succinate (d), -fumarate (e), -malate (f), and -citrate (h) *via* the Krebs cycle (Figs. S2*B* and 1*B*). ^13^C_4_-malate can be converted to ^13^C_3_-pyruvate (l) *via* the ME reaction, leading to the synthesis of ^13^C_2_- and ^13^C_3_-citrate, -succinate, -fumarate, -malate, and -Asp *via*, respectively, PDH- and PC-initiated Krebs cycle activities. Moreover, ^13^C_x_,^15^N-Asp (j) can be produced *via* GOT-catalyzed transamination while ^13^C_x_,^15^N-GSH (m) is synthesized from ^13^C_x_,^15^N-Glu. Such pathway reconstruction was deduced from the presence of all expected ^13^C and ^13^C,^15^N-isotopologues of the glutaminolytic and Krebs cycle products based on the MS^1^ and MS^2^ data. Arsenite transformed cells (BAsT) showed depletion of all of these products except for the labeled GSH in terms of both Glu (n) and Gly (o) moieties (Fig. 1*B*). These data pointed to inhibition of the glutaminase and/or Krebs cycle activity but activation of GSH synthesis in BAsT *versus* control cells.

In addition, detailed analysis of the ^13^C- and/or ^15^N-labeling patterns of both the parent metabolites (molecular ions in MS^1^) and fragments (in MS^2^) revealed differential arsenite effects on individual enzyme reactions. For example, the first two products of glutaminase (*i.e.*, ^13^C_5_,^15^N-Glu in a and ^13^C_5_-αKG in b) showed arsenite-induced depletion, which suggests glutaminase inhibition by arsenite. However, from the MS^2^ data, we saw ^13^C_3_ (3)- and ^13^C_4_ (4)-C1 to C5-citrate (i) accumulated while the product ^13^C_3_ (3)- and ^13^C_4_ (4)-C1 to C4-αKG depleted (c), which points to additional block at the aconitase and/or isocitrate dehydrogenase steps. The former is consistent with the known inhibition of aconitase by arsenite (24). If this were the only effect of arsenite, we would expect the same trend for the MS^1^ data for citrate (h), which was not the case. The production of these fragments had a contribution from the ME (, light blue box) and/or PC (, green box) in addition to the glutaminase (, red box)-mediated pathways. The observed discrepancy between MS^1^ and MS^2^ data could be attributed to the confounding activation of the ME and PC-mediated pathways by arsenite, leading to the accumulation of the three citrate fragments. This interpretation could also apply to the discrepancy between MS^1^ (f) and MS^2^ (g) data of malate. The accumulation of ^13^C_4_-succinate (d) and depletion of the products ^13^C_4_-fumarate (e) are consistent with the inhibition of succinate dehydrogenase (SDH) based on the MS^1^ data, which was reported previously (25). Moreover, the arsenite-induced accumulation in the ^13^C_5_, ^15^N_1_-Glu (n), and ^13^C_2_, ^15^N_1_-Gly moieties (o) of GSH argue for the activation of the GSH synthesis pathway while that in ^15^N_1_-Glu suggests enhanced GOT activity in addition. The former is consistent with arsenite-induced GSH accumulation and activation of GSH synthesis genes reported for lung epithelial cells (25). Thus, by combining the MS^1^ and MS^2^ data, it is practical to translate changes in the complex ^13^C- and ^15^N-labeling patterns of the Krebs cycle metabolites into altered activity of specific enzymes, which would not be reliable based on either MS1 or MS2 data alone.

### The PPP and gluconeogenesis

**Figure 2:**
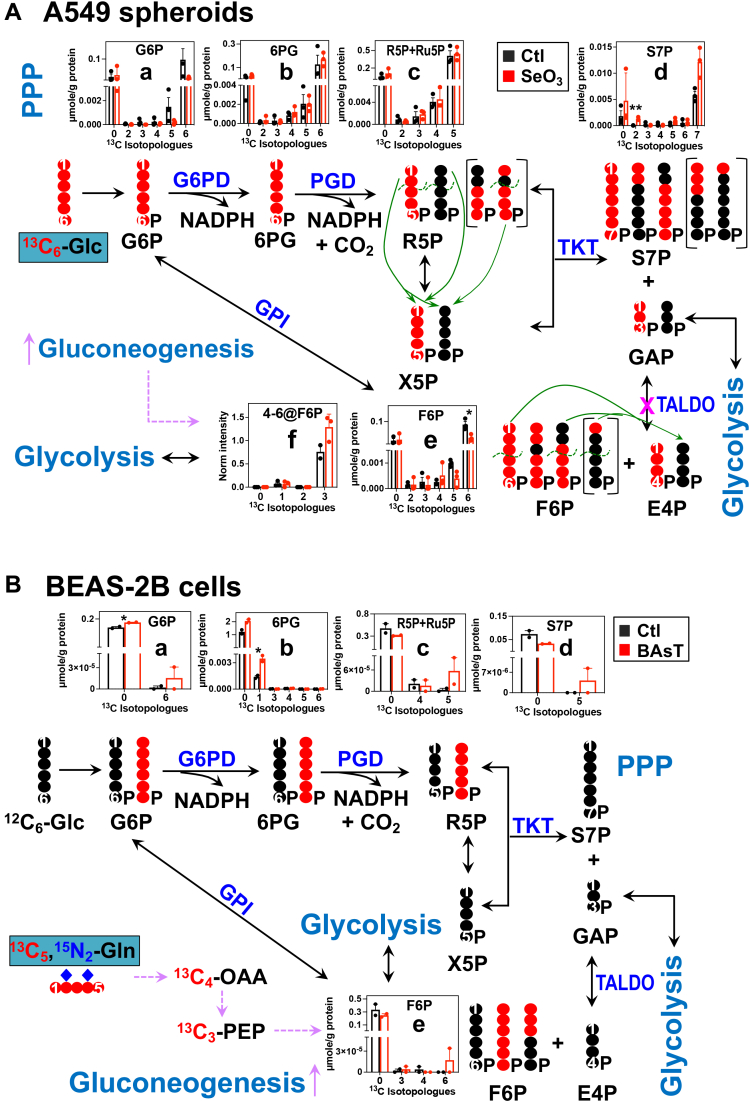
^**13**^**C isotopologue analysis of IC-UHR-MS**^**1**^**and MS**^**2**^**data shows enhanced oxidative PPP in response to selenite in A549 spheroids or to arsenite transformation in BEAS-2B cells.***A*, A549 spheroids. *B*, BEAS-2B cells. ^13^C atoms were traced from [^13^C_6_]-Glc (*A*) or [^13^C_5_,^15^N_2_]-Gln (*B*) into the PPP and gluconeogenic products. *Brackets* in (*A*) denote ^13^C products of the reverse transaldolase (TALDO) reaction, while *green curves* and *arrows* delineate the recombination of R5P moiety with X5P (TKT reaction) or F6P moiety with E4P (TALDO reaction) to generate sedoheptulose-7-phosphate (S7P). Due to space limitation, not all possible labeled products are shown. The same sets of extracts as in Figure 1 were analyzed by IC-UHR-MS^1^ and DI-MS^2^. The X-/Y-axes and number of replicates are as in Figure 1. 6PG, 6-phosphogluconate; E4P, erythrose-4-phosphate; G6PD/PGD, glucose-6-phosphate/6-phosphogluconate dehydrogenase; GPI, glucose-6-phosphate isomerase; PPP, pentose phosphate pathway; R5P, ribose-5-phosphate; Ru5P, ribulose-5-phosphate; S7P, sedoheptulose-7-phosphate; TK, transketolase; X5P, xylulose 5-phosphate; all other abbreviations and symbols are as in Fig. S2. ∗, *p* < 0.05; ∗∗, *p* < 0.01.

The PPP is a major route for glucose oxidation to produce ribose-5-phosphate (R5P) and NADPH, which are respectively the precursor to nucleotide synthesis and reductant for anabolic and antioxidant metabolism. In this pathway, [^13^C_6_]-Glc is converted to ribulose-5-phosphate (Ru5P) *via* hexokinase, G6P dehydrogenase, and 6-phosphogluconate dehydrogenase, which is then isomerized to R5P (oxidative branch) and epimerized to xylulose-5-phosphate, followed by the transketolase (TKT) and transaldolase (TALDO) reactions to respectively produce sedoheptulose-7-phosphate (S7P) + glyceraldehyde-3-phosphate and fructose-6-phosphate (F6P) and erythrose-4-phosphate (freely reversible nonoxidative branch), respectively (Fig. 2*A*). In [^13^C_6_]-Glc–traced A549 cells, we saw domination of fully ^13^C-labeled isotopologues of G6P (a), 6PG (b), Ru5P/R5P (c), and S7P (d) in the MS^1^ data (Fig. 2*A*). For S7P, the ^13^C_2_- and ^13^C_5_-isotopologues were also present and at higher levels than the ^13^C_1_- (absent) and ^13^C_3_-isotopologues. Based on the TKT and TALDO reaction mechanism (denoted by green arrows), the former two species can be produced directly by the forward TKT reaction and the latter two species by the reverse TALDO reaction. Thus, the observed scrambled ^13^C-labeling patterns of S7P is consistent with higher forward or oxidative PPP than reverse or nonoxidative PPP activity. Selenite treatment enhanced the levels of ^13^C_2_- and ^13^C_5_-S7P while reducing those of ^13^C_1_- and ^13^C_3_-S7P (d), which suggests a shift from nonoxidative to NADPH-generating oxidative PPP. This is consistent with the lack of depletion of ^13^C-6PG (b) and ^13^C-R5P+Ru5P (c) despite the large depletion of G6P (a) by selenite. Interestingly, selenite induced depletion of ^13^C_5_- and ^13^C_6_-F6P (e) but buildup of the ^13^C_3_-4,5,6 fragment of F6P (f). Together with the accumulation of ^13^C-labeled S7P, the former points to inhibition of TALDO activity by selenite while the latter could be attributed to enhanced gluconeogenesis by selenite (*cf.*, Fig. S3).

In [^13^C_5_,^15^N_2_]-Gln–traced BEAS-2B cells, very low levels of ^13^C incorporation were evident in some of the PPP products and their ^13^C scrambling patterns presumably resulted from a combination of gluconeogenic, TKT, and TALDO activities (Fig. 2*B*). The fully ^13^C-labeled isotopologues of G6P (a), R5P+Ru5P (c), and F6P (e) as well as ^13^C_1_-6PG (b) accumulated more in BAsT than control cells. Although most of these changes were at the detection limit and nonstatistically significant, they could reflect enhanced oxidative PPP activity in BAsT cells (*cf.*, Fig. 2*A*). This would generate more NADPH to support reduction of GSSG to GSH (*cf.*, Fig. 1*B*) for relieving oxidative stress induced by arsenite (25).

### UDP-GlcNAc biosynthesis pathway

**Figure 3:**
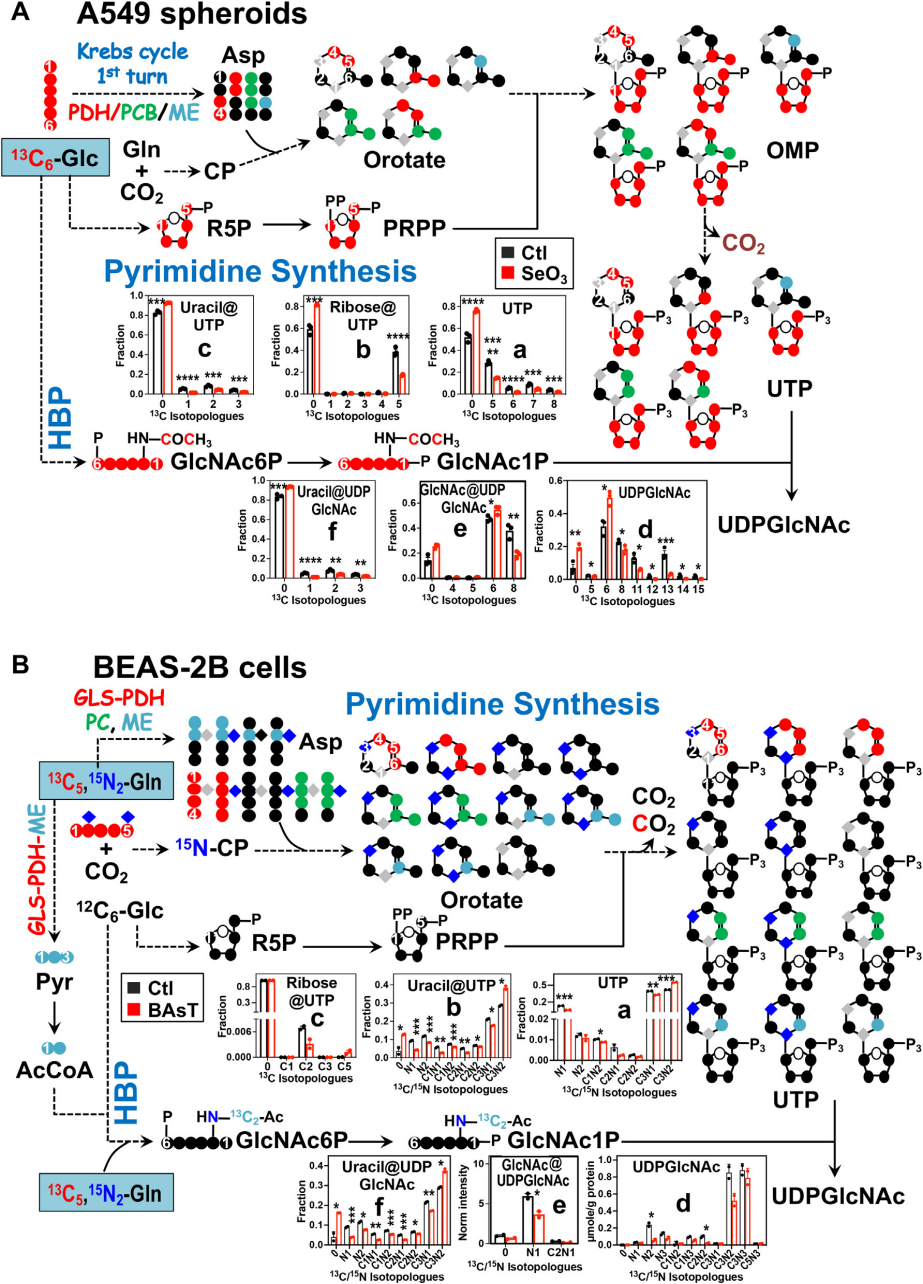
**Altered**^**13**^**C and/or**^**15**^**N incorporation into UTP/UDP-GlcNAc and their moieties in response to selenite in A549 spheroids or to arsenite transformation in BEAS-2B cells.***A*, A549 Spheroids. *B*, BEAS-2B cells. ^13^C and/or ^15^N atoms were traced from [^13^C_6_]-Glc (*A*) or [^13^C_5_,^15^N_2_]-Gln (*B*) into UDP-GlcNAc. ●: ^12^C; : ^14^N; : ^15^N; , , : ^13^C from the first turn of the PDH, PCB, and ME-mediated Krebs cycle reactions, respectively. The same sets of extracts as in Figure 1 were analyzed by IC-UHR-MS^1^ and DI-MS^2^. *A*-a, *B*-a and *A*-d, *B*-d: determined from MS^1^ of UTP/UDP-GlcNAc; A-b to c/f and B-b to c/f: determined from the MS^2^ of the ribose and uracil moieties of UTP and UDP-GlcNAc in A549 spheroid and BEAS-2B cells, respectively; *A*-e and *B*-e: determined from the MS^2^ of the GlcNAc moiety of UDP-GlcNAc in A549 spheroid and BEAS-2B cells, respectively. The X-/Y-axes and number of replicates are as in Figure 1. Ac, acetyl; CP, carbamoyl phosphate; GlcNAc1 or 6P, N-acetylglucosamine 1 or 6-phosphate; GLS, glutaminase; HBP, hexosamine biosynthesis pathway; ME, malic enzyme; OMP, orotidine monophosphate; PC, pyruvate carboxylase; PDH, pyruvate dehydrogenase; Pyr, pyruvate. ∗*p* < 0.05; ∗∗*p* < 0.01; ∗∗∗*p* < 0.005; ∗∗∗∗*p* < 0.001; ∗∗∗∗∗*p* < 0.0005.

UDP-GlcNAc is an activated form of GlcNAc needed for O- and N-linked protein glycosylation, which are important in regulating numerous cellular processes, such as protein targeting to organelles (26) and nutrient sensing (27, 28). UDP-GlcNAc has four biochemical moieties (Fig. S4) that are derived from several intersecting metabolic pathways (29) (Fig. 3). The hexosamine moiety comes from glucose and the amido N of Gln *via* the hexosamine biosynthesis pathway (HBP), the acetyl group is donated from acetyl CoA generated from glucose, amino acids, or fatty acids, the ribose unit derives from glucose *via* the PPP, and the uracil ring is produced from pyrimidine biosynthesis using C and N sources such as glucose and Gln.

From the UHR-MS^1^ spectra of UDP-GlcNAc, high intensity of the ^13^C_6_-, ^13^C_8_-, ^13^C_11_-, and ^13^C_13_-peaks were observed in [^13^C_6_]-Glc–traced A549 cells (Fig. 3*A*-d). The ambiguities in the labeled unit assignment for these isotopologues were resolved with the DI-MS^2^ data. We observed low enrichment of ^13^C_1-3_ (1–3) peaks in the uracil fragment of UDP-GlcNAc (Fig. 3*A*-f), which was akin to the corresponding pattern of the precursor UTP (Fig. 3*A*-c). In contrast, the glucosamine plus acetyl fragment showed high enrichment of the ^13^C_6_ (6) and ^13^C_8_ (8) species (Fig. 3*A*-e), as the case for the two in the MS^1^ data (Fig. 3*A*-d). These two species can be confidently assigned to ^13^C_6_-glucosamine- and ^13^C_6_-glucosamine- + ^13^C_2_-acetyl–bearing UDP-GlcNAc, respectively. Although we did not directly observe relevant fragments, we can justifiably assign two other abundant isotopologues (^13^C_11_ and ^13^C_13_) to, respectively, ^13^C_6_-glucosamine + ^13^C_5_-ribose- and ^13^C_6_-glucosamine- + ^13^C_5_-ribose + ^13^C_2_-acetyl–bearing UDP-GlcNAc, based on the prevalence of the ^13^C_6_-glucosamine and ^13^C_6_-glucosamine + ^13^C_2_-acetyl moieties (Fig. 3*A*-e) as well as ^13^C_5_-ribose in the UTP precursor (Fig. 3*A*-b). Selenite treatment enhanced the enrichment of the ^13^C_6_-GlcNAc fragment of UDP-GlcNAc (e) but reduced that of the ^13^C_8_-GlcNAc (e) and ^13^C_1-3_-uracil fragments of UDP-GlcNAc (f) as well as the ^13^C_8, 11-15_ isotopologues of intact UDP-GlcNAc (d) (Fig. 3*A*). These data are consistent with the block in the uracil synthesis plus reduced synthesis and/or incorporation of ribose into UTP and UDP-GlcNAc, as either or both processes are required for the synthesis of the ^13^C_11-15_-isotopologues. They also point to the maintenance of glucosamine synthesis but reduced acetyl incorporation into GlcNAc *via* the HBP. Again, such detailed deduction of selenite’s effect on the UDP-GlcNAc biosynthetic pathway would not be feasible without the combined MS^1^ and MS^2^ data.

In [^13^C_5_,^15^N_2_]-Gln–traced BEAS-2B cells, UHR-MS^1^ data of UDP-GlcNAc showed isotopologues with single (^15^N) and dual (^13^C,^15^ N) tracer atoms (Fig. 3*B*-d). Together with the MS^2^ fragment data, the two most abundant ^13^C_3_,^15^N_2_ (C3N2) and ^13^C_3_,^15^N_3_ (C3N3) species in the MS^1^ data mainly consisted of ^13^C_3_,^15^N_1_- and ^13^C_3_,^15^N_2_-uracil (e) plus ^15^N_1_-glucosamine units (f), respectively, with minor contribution of the ^13^C_1_,^15^N_1_-uracil plus ^13^C_2_,^15^N_1_-glucosamine unit. This is consistent with the prominence of the ^13^C_3_,^15^N_1_- and ^13^C_3_,^15^N_2_-uracil fragment in the UTP precursor (b). As illustrated in the atom-resolved pathway scheme, these two most abundant species should be derived from the reaction sequence of glutaminase—first turn of the Krebs cycle (PDH, PC, ME-mediated)—pyrimidine synthesis. The MS^2^ fragment of GlcNAc showed dominant enrichment of the ^15^N_1_-species with some enrichment of the ^13^C_2_,^15^N_1_-species (f) (Fig. 3*B*). These data indicate high activity of HBP along with ME-mediated Krebs cycle reactions giving rise to ^13^C_2_-acetyl CoA for acetyl transfer to glucosamine (*cf.*, Fig. S4). Arsenite transformation depleted the low-abundance ^15^N and ^13^C,^15^N-isotopologues of UDP-GlcNAc (d) and its precursor UTP (a), which primarily resulted from reduced ^15^N and/or ^13^C incorporation into the GlcNAc and uracil moieties (b,e,f) since little ^13^C enrichment was evident in the ribose unit of UTP (c) (Fig. 3*B*). Thus, chronic exposure to arsenite blocked both HBP and uracil biosynthesis in BEAS-2B cells. However, the enrichment of the most abundant ^13^C_3_,^15^N_2_-uracil fragment of UDP-GlcNAc and its precursor UTP was enhanced by arsenite (b,e). This species can be derived from ^13^C_4_,^15^N_1_-Asp (with loss of ^13^CO_2_) + ^15^N-carbamoyl phosphate (^15^N-CP). As the ^13^C_3_,^15^N_1_-uracil fragment of both UTP and UDP-GlcNAc was reduced in enrichment, it is plausible that enhanced enrichment of ^13^C_3_,^15^N_2_-uracil–bearing UDP-GlcNAc is driven by the formation and/or incorporation of ^15^N-CP at the expense of the ^13^C_3_,^15^N_1_ species in arsenite-transformed BEAS-2B cells. Such detailed deduction of pathway changes is made possible by the use of the dual tracer in combination with the ability to resolve label positions in UDP-GlcNAc moieties by the DI-MS^2^ method.

## Discussion

We have applied a previously developed Ion chromatography-ultrahigh resolution Fourier transform MS^1^/DI-MS^2^ method (21) for extensive and robust reconstruction of [^13^C_6_]-Glc or [^13^C_5_, ^15^N_2_]-Gln–fueled central metabolic networks in mammalian cells. This method met the needs for resolving dual tracer distribution in intact metabolites with ultra high-resolution MS^1^ while simultaneously acquiring positional labeling in metabolite moieties *via* DI-MS^2^. In this report, we illustrated how to rigorously reconstruct the Krebs cycle, PPP, gluconeogenesis, and UDP-GlcNAc synthesis pathway by utilizing the combination of UHR-MS^1^ with MS^2^ data. This approach enabled us to unambiguously discern *in-cell*–altered activity of specific enzymes induced by anticancer selenite treatment in lung adenocarcinoma A549 spheroids or by arsenite transformation in lung epithelial BEAS-2B cells.

For A549 spheroids, we found that selenite’s ability to attenuate the Krebs cycle activity lies in the blockade of enzymes both in the canonical (OGDH) and anaplerotic (PC, ME) pathways (Fig. 1*A*). This is consistent with the suppression of the *OGDH* gene and PC protein but contrary to the overexpression of the *ME* gene in the 2D counterparts reported previously (13, 30). Another notable distinction of selenite’s effect is less inhibition of GSH synthesis in 3D (Fig. 1*A*) *versus* 2D A549 cells (15), which should contribute to a better capacity of the spheroid culture for antioxidation. Our present data points to reduced synthesis (*i.e.*, blocked GOT), rather than attenuated incorporation, of the precursor Glu as the cause for selenite’s inhibition of GSH synthesis in A549 spheroids. This is reasoned from the depletion of ^13^C-labeled Glu despite the buildup of its ^13^C-labeled αKG precursor. As for PPP, selenite-induced shift to the oxidative branch is expected to produce more NADPH to better sustain the reduction of GSSG to GSH, which is used to alleviate oxidative stress by detoxifying reactive oxygen species (15). This shift can also maintain R5P production despite the block of the TALDO activity in the nonoxidative branch (Fig. 2*A*). These changes of the GSH and R5P synthesis pathways in 3D A549 spheroids presumably contribute to their better resistance to selenite toxicity than the 2D counterpart, as observed previously (15). In addition, our combined MS^1^ and MS^2^ data revealed that subsequent R5P incorporation into UTP and the supply of acetyl CoA and/or its entry into HBP was blocked by selenite, leading to attenuated synthesis of UDP-GlcNAc. This, together with somewhat compromised Krebs cycle, could underlie the growth inhibition of A549 spheroids with prolonged selenite treatment (15).

Arsenite is known to impact various metabolic proteins that contain the sulfhydryl group (31) (*e.g.*, IκB kinase and glucose transporter) leading to different disease states including cancer (32, 33). However, the details of metabolic reprogramming in transformed epithelial cells induced by chronic, low-dose exposure to arsenite are still elusive. Our MS^1^- and MS^2^-based metabolic network reconstruction revealed the complex action of arsenite on the Krebs cycle, PPP, and antioxidation pathways in lung epithelial BEAS-2B cells, including blockade of aconitase, isocitrate dehydrogenase, SDH, and glutaminase but activation of ME/PC, GOT, and GSH synthesis activities. One important outcome of these reprogrammed events can be reactive oxygen species buildup but not in excess to avoid apoptosis while driving different carcinogenic events (33). Moreover, despite the block of HBP and overall uracil synthesis, arsenite-transformed BEAS-2B cells largely maintained UDP-GlcNAc production by activating the CP synthesis and/or incorporation steps of the UDP-GlcNAc synthesis pathway. UDP-GlcNAc is the required substrate for O-GlcNAcylation of several oncogenic regulators that drive cancer development (34) and the maintenance of this oncometabolite pool is expected to be important to arsenite transformation of BEAS-2B cells.

In conclusion, we applied an IC-UHR-MS^1^/DI-MS^2^ method to track changes in ^13^C/^15^N-labeling patterns of metabolites and their moieties in SIRM studies of A549 spheroids or BEAS-2B cells in response to selenite or arsenite transformation, respectively. This approach enabled robust reconstruction of the metabolic network consisting of the Krebs cycle, PPP, gluconeogenesis, and UDP-GlcNAc synthesis pathway to discern specific enzyme activities in the network altered by the treatments. In turn, this information helps elucidate the resistance mechanism of 3D *versus* 2D A549 cultures to selenite and metabolic reprogramming that can mediate the transformation of BEAS-2B cells by arsenite.

## Experimental procedures

### Materials

All materials including the make-up solvent methanol for Ion chromatography, individual standards of metabolites used for quantification were obtained as described previously (21).

### Preparation of calibration standard mixtures

A mixture of 86 (Mix 1) and 81 (Mix 2) standards were prepared as two separate calibration standard mixtures as described previously (21). The standard mixtures were aliquoted, lyophilized, and stored at −80 °C for long term use. When needed, lyophilized Mix 1 was dissolved in 120 μl 18 MΩ water, vortexed, and 50 μl was used to reconstitute with Mix 2 to form the final calibration standard mixture.

### IC-UHR-MS^1^ and DI-MS^2^

#### Ion chromatography-ultrahigh resolution fourier transform MS

Metabolites were separated on an IonPac AG11-HC-4 μm guard column (2 × 50 mm) coupled to an IonPac AS11-HC-4 μm RFIC&HPIC (2 × 250 mm) analytical column in a Dionex ICS5000^+^ system (Thermo Scientific) equipped with a dual pump, an eluent generator, an autosampler, and a detector/chromatography module. Conditions for chromatographic separations (*i.e.*, KOH gradient) and ion suppressor and desolvation in the heated electrospray were as described previously (21). MS data were acquired using the Xcalibur software. A batch of samples started with a 15 min blank (water) injection to check for contamination in the instrument, followed by two injections of calibration standard mixtures to ensure the stability of MS signals and another 15 min water injection to check for carryover on the IC column. Lyophilized cell extracts were freshly reconstituted in 20 μl 18 MΩ water plus 1 μM DSS (sodium trimethylsilylpropanesulfonate) and run in a random order. Each sample was followed by one or two 15 min injections of water blank to minimize carryover. The calibration standard mixture was run after every 6 to 8 cell extracts to track signal loss in the same batch of run. Each sample batch ended with an injection of the calibration standard mixture, followed by water to double check the normality of MS signals and sample carryover.

#### DI-MS^2^ measurement for cell polar extracts

DI-MS^2^ analysis was performed in between full MS^1^ scans for quantifying targeted fragment(s) of major metabolites in polar extracts, as described previously (21). To achieve this, we set (1) the cycle time of no more than 2 to 3 s for acquiring 10 to 15 points across each chromatographic peak for reliable quantification of precursors and their isotopologues; (2) sufficient resolving power in full scan (500,000) and MS^2^ (60,000) modes to discriminate ^13^C from ^15^N-containing isotopologues of precursors and fragments; and (3) full isotopologue coverage for each metabolite in selecting the precursor mass range for MS^2^ scan (*i.e.*, 280–440 with the isolation window of 200 *m/z*). Other conditions were as described previously (21).

### Data analysis and quantification

We first established an in-house exact mass database for the precursors and fragment products based on the corresponding mass ion spectra acquired for individual metabolite standards. Several public metabolomics databases, including the Human Metabolome DataBase (35), the Kyoto Encyclopedia of Genes and Genomes (36), and METLIN (37), and Mass Frontier were used to help interpret MS^2^ data for metabolite fragmentation patterns. This database was then incorporated into TraceFinder v3.3 (Thermo Scientific) for assigning and integrating the peak areas of precursor ions in MS^1^ spectra and fragment ions in MS^2^ spectra of targeted metabolites in cell extracts for further quantification. Precursors and fragments were assigned with mass accuracy set to 5 ppm. Assignments were curated before isotopic peak areas were corrected for natural abundance as previously described (38). Metabolites in samples were quantified from the corrected MS^1^ data by calibrating against the two calibration standard mixtures run before (Std 1) and after (Std 2) the samples. The response factor was calculated for each sandwiched sample run as follows:

Response factor = (Area [Std 1] + (Area [Std 2] – Area [Std 1]) × nth run number/run number))/std concentration. The metabolite concentration was then calculated by dividing the corrected MS^1^ peak area with the response factor and normalized against the extract aliquot and amount of total protein. The fragment peak areas were similarly normalized.

### Preparation of ^13^C-labeled polar extracts of 3D A549 spheroids ± selenite

A549 cells were grown to 90% confluence in 10-cm plates, followed by loading with magnetic nanoparticles (Nanoshuttle, N3D Biosciences) overnight at 37 °C/5% CO_2_, as described previously (15). Cells were then detached and seeded into 6-well Costar-cell repellent plates (Corning, Inc) at 400,000 cells/well for spheroid formation. Spheroids were cultured for 4 days before medium change to [^13^C_6_]-Glc ± 10 μM Na_2_SeO_3_ and grown at 37 °C/5% CO_2_ for 24 h. Spheroids were harvested, rinsed twice with cold PBS, and then briefly with cold nanopure water before simultaneous quenching and extraction of polar metabolites in cold 70% methanol (15). One-eighth of the polar fraction was aliquoted and lyophilized for IC-UHR-MS^1^/DI-MS^2^ analysis.

### Preparation of ^13^C-, ^15^N-labeled polar extracts of 2D BEAS-2B cells ± arsenite transformation

Primary bronchial epithelial BEAS-2B cells (ATCC) were cultured under two conditions: (1) in Bronchial Epithelial Cell Growth Medium (BEGM, Lonza Corporation) as control; (2) in BEGM + 1 μM Na_2_AsO_3_ in 10-cm plates as transformed cells (BAsT). Cells were grown to 60 to 70% confluence before passaging to generate over 24 weeks. At week 24, 4 mM [^13^C_5_,^15^N_2_]-Gln was introduced to both groups and grown at 37 °C/5% CO_2_ for 24 h. Cells were then quenched with cold acetonitrile and extracted for polar metabolites in acetonitrile/water/chloroform (V/V 2:1.5:1) as described previously (16, 39). One-eighth of the polar fraction was aliquoted and lyophilized for IC-UHR-MS^1^/DI-MS^2^ analysis.

## Data availability

All data acquired are available upon request.

## Supporting information

This article contains supporting information (40, 41).

## Conflict of interest

The authors declare that they have no conflicts of interest with the contents of the article.
