# Supplementary material for: Ultrahigh resolution MS1/MS2-based reconstruction of metabolic networks in mammalian cells reveals changes for selenite and arsenite action
Source: J Biol Chem. 2022 Oct 9;298(12):102586. doi: 10.1016/j.jbc.2022.102586 (PMC9667311; doi:10.1016/j.jbc.2022.102586)
Supplement: Supplemental Figures S1–S4 [file mmc1.pdf]

Ultrahigh resolution MS[1]/MS[2]-based Reconstruction of Metabolic Networks in Mammalian Cells

Teresa W-M. Fan[1,2,3], Qiushi Sun[1#], Richard M. Higashi[1,2,3]

Supporting information (Figures S1, S2, S3, and S4)

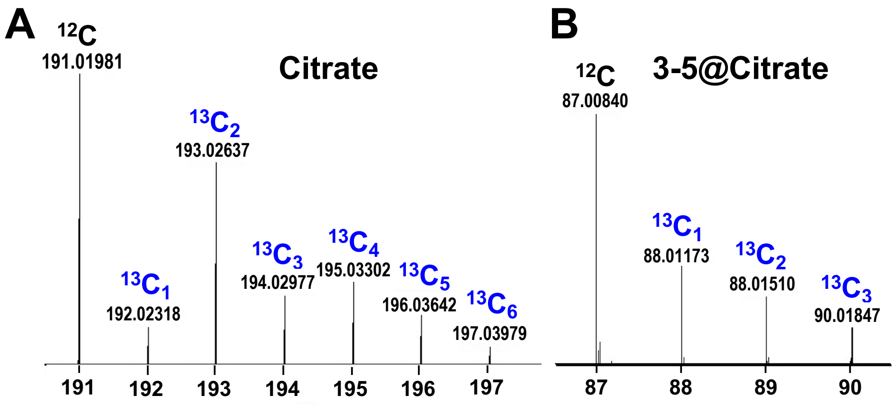

Figure S1. Example MS^1 and MS^2 spectra of citrate in A549 spheroid extracts.
The two UHR-FTMS spectra were acquired from a control A549 spheroid extract in **Fig. 1A**. The isotopologue species of citrate were assigned based on the chromatographic retention time and accurate mass. ^{12}C represent the all ^{12}C (0) isotopologue. **A**: MS^1 spectrum of the intact citrate molecule; **B**: MS^2 spectrum of the $^{13}C_3$-3,4,5-citrate fragment.

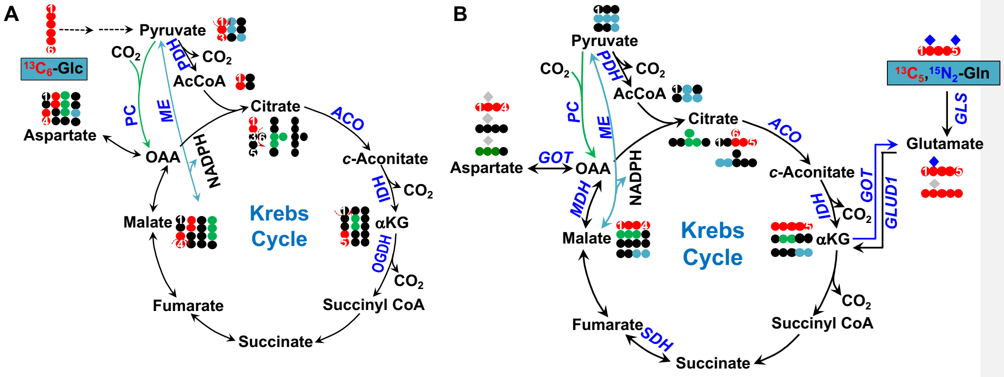

Figure S2. ^{13}C atom tracing for [$^{13}C_6$]-glucose and [$^{13}C_5$,$^{15}N_2$]-Gln transformations in the Krebs cycle

The pathway scheme in **A** tracks the transformations of [$^{13}C_6$]-glucose into various Krebs cycle metabolites in terms of the number and position of the ^{13}C atom (●/●/●). For example, the condensation of $^{13}C_2$-1,2-acetyl CoA (AcCoA, ●●) with preexisting unlabeled oxaloacetate (OAA, ●●●●) produces $^{13}C_2$-1,2-citrate (●●●●●●), which with the loss of CO_2 at C6 and inversion of the molecular structure at the aconitase (ACO) step (1) generates $^{13}C_2$-4,5-α–ketoglutarate (αKG, ●●●●●). Subsequent loss of another CO_2 from αKG at C1 via the oxoglutarate dehydrogenase (OGDH) reaction generates $^{13}C_2$-1,2- (●●●●) or $^{13}C_2$-3,4-succinate (●●●●) and subsequently $^{13}C_2$-3,4-+$^{13}C_2$-1,2-fumarate and malate. Such label scrambling occurs due to the structural symmetry of succinate. Carboxylation of $^{13}C_3$-1,2,3-pyruvate (●●●) at C3 by pyruvate carboxylase (PC) leads to the synthesis of $^{13}C_3$-1,2,3-Asp (●●●●) while loss of CO_2 at C4 from $^{13}C_2$-3,4-malate via the malic enzyme (ME) reaction produces $^{13}C_1$-3-pyruvate (●●●). Likewise, the scheme in **B** traces the transformations of [$^{13}C_5$,$^{15}N_2$]-Gln into various Krebs cycle metabolites in terms of the number and position of the ^{13}C atom (●/●/●). Deamidation of labeled Gln produces $^{13}C_5$,$^{15}N_1$-Glu, which is deaminated to $^{13}C_5$-αKG (●●●●●) to enter the Krebs cycle. Subsequent Krebs cycle reactions generate $^{13}C_4$-malate (●●●●), which undergoes ME and pyruvate dehydrogenase (PDH) reactions to produce $^{13}C_3$-pyruvate (●●●) and $^{13}C_2$-AcCoA, respectively. $^{13}C_3$-pyruvate (●●●) is then transformed via the Krebs cycle reactions to produce $^{13}C_2$-1,2-citrate (●●●●●●), $^{13}C_2$-4,5-αKG (●●●●●), and $^{13}C_2$-3,4-malate (●●●●). $^{13}C_3$-pyruvate can also be carboxylated by PC to produce $^{13}C_3$-1,2,3-malate/-Asp (●●●●) and $^{13}C_3$-3,4,6-citrate (●●●●●●). Red arrows depict cleavage of carbon-carbon bonds. Not all reaction products are shown. ●: ^{12}C; ◆: ^{14}N; ◆: ^{15}N. See **Fig. 1** for all other abbreviations.

Commented [QS1]: This labeling pattern isn't shown in B.

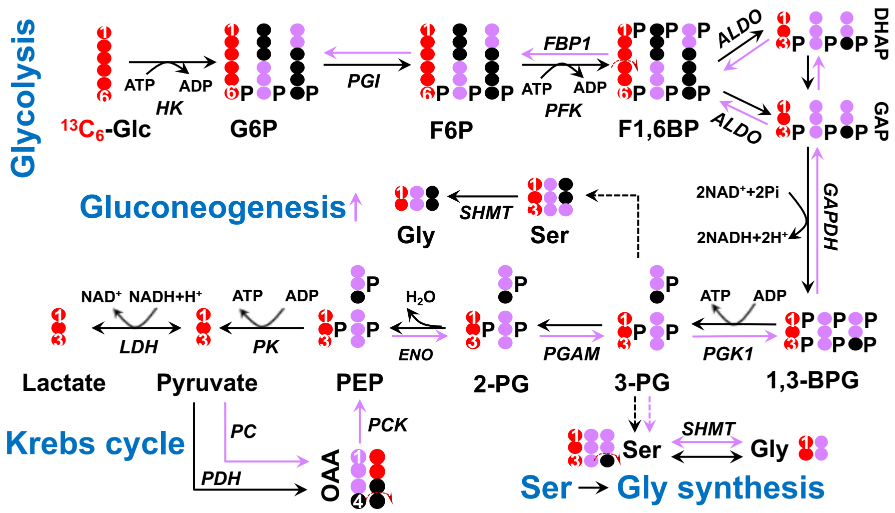

Figure S3. ^{13}C atom tracing for [$^{13}C_6$]-glucose transformations via glycolysis, Ser→Gly synthesis, and gluconeogenesis pathways.

[$^{13}C_6$]-glucose is converted to fructose-1,6-bisphosphate (F1,6BP, ●●●●●●) via phosphorylation and isomerization , which is cleaved between C3 and C4 to generate $^{13}C_3$-dihydroxyacetone-3-phosphate (DHAP) and $^{13}C_3$-glyceraldehyde-3-phosphate (GAP) (●●●). Subsequent glycolytic reactions generate $^{13}C_3$-pyruvate, which in turn produce $^{13}C_2$-1,2-oxaloacetate (OAA, ●●●●) and $^{13}C_3$-1,2,3-OAA (●●●●) via PDH- or PC-initiated Krebs cycle reactions. The two labeled OAA species then undergo the first step of gluconeogenic (PCK) reaction to produce $^{13}C_3$-PEP (●●●) and $^{13}C_1$-1,2-PEP (●●●), which lead to the synthesis of $^{13}C_3$-4,5,6-F1,6BP (●●●●●●) and $^{13}C_1$-1,2-F1,6BP (●●●●●●) via subsequent gluconeogenic reactions. The glycolytic product 3-phosphoglycerate (3-PG ●●●) is the precursor to Ser (●●●) and in turn Gly (●●) synthesis. Not all reaction products are shown. ● represents ^{12}C and ●/● refer to ^{13}C derived from glycolysis/gluconeogenesis respectively. Solid and dash arrows indicate single- and multi-step reactions. Red arrows depict cleavage of carbon-carbon bonds. HK: hexokinase; PGI: phosphoglucose isomerase; PFK: phosphofructose kinase; FBP1: fructose-bisphosphatase 1; ALDO: fructose-bisphosphate aldolase; GAPDH: glyceraldehyde-3-phosphate dehydrogenase; PGK1: phosphoglycerate kinase 1; PGAM: phosphoglycerate mutase; ENO: enolase; PK: pyruvate

kinase; LDH: Lactate Dehydrogenase; PCK: phosphoenolpyruvate carboxykinase; PC: pyruvate carboxylase; SHMT: serine hydroxymethyltransferase.

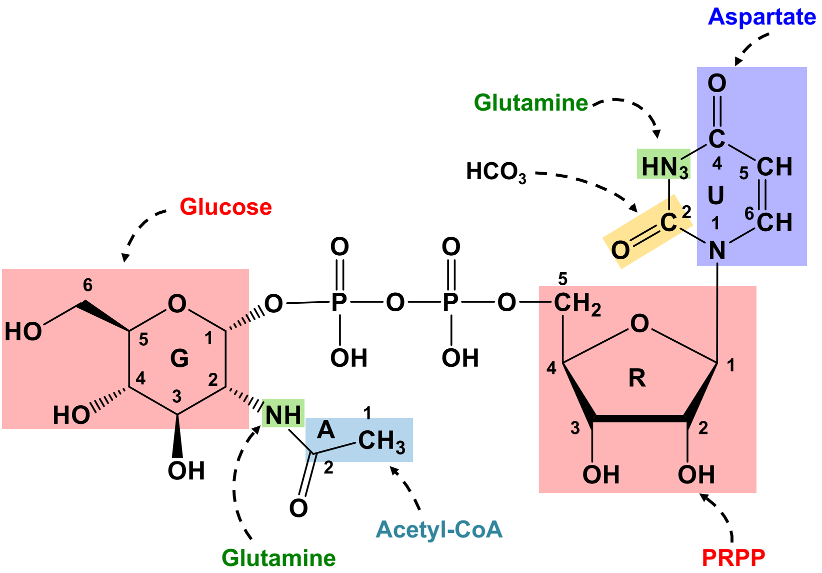

Figure S4. Sources of carbon and nitrogen atoms for the synthesis of UDP-GlcNAc.
The diagram is a copy of **Fig. 1** in (2). Atoms are numbered according to the international convention in individual subunits. G, A, R, and U represent the glucose, acetyl, ribose, and uracil subunits, respectively. UDP-GlcNAc: uridine diphosphate-N-acetylglucosamine; PRPP: phosphoribosyl pyrophosphate.

References

1. Lloyd, S. J., Lauble, H., Prasad, G. S., and Stout, C. D. (1999) The mechanism of aconitase: 1.8 A resolution crystal structure of the S642a:citrate complex. *Protein Sci* **8**, 2655-2662
2. Sun, Q., Fan, T. W. M., Lane, A. N., and Higashi, R. M. (2020) Applications of chromatography-ultra high-resolution MS for stable isotope-resolved metabolomics (SIRM) reconstruction of metabolic networks. *TrAC Trends in Analytical Chemistry* **123**, 115676
